# Real-World Challenges of Promoting Community Health

**Published:** 2007-06-15

**Authors:** Pattie Tucker, Amanda M Navarro

**Affiliations:** Division of Adult and Community Health, National Center for Chronic Disease Prevention and Health Promotion, Centers for Disease Control and Prevention; Public Health Division, Northrop Grumman Corporation, Atlanta, Ga

Over the past 30 years health promotion efforts have been targeted at the local level, recognizing that this is the locus of innovative, grassroots efforts to improve the health of the community. Yet addressing the realities of working with local communities is considered to be outside the purview of the public health field; therefore, community-level efforts are ignored. Although we support the recommendations made by the National Expert Panel on Community Health Promotion (1), there are several challenges to be faced in accomplishing these goals.

One recommendation made by the expert panel was to *promote community-based participatory research (CBPR) within and outside of CDC* (Centers for Disease Control and Prevention). This approach emphasizes community participation at all stages of research and program development, but there are imbalances of power that cannot be dismissed (2). For example, past abuses by researchers, particularly in communities of color, have led many community organizations to act as "gatekeepers" to protect their constituents. Lack of access to these communities will make it difficult to estimate disease burden and to improve the overall health of the community. Another restriction on community health promotion efforts is the power dynamics between communities and groups such as businesses and policy makers. Initiatives targeting policy and environmental change (e.g., improving access to fruits and vegetables in local bodegas and establishing large grocery stores in impoverished communities) are highly encouraged through programs such as the Racial and Ethnic Approaches to Community Health (REACH) and the Prevention Research Centers. However, many small communities must confront marketing giants such as national food or drink corporations to make a community-wide impact. CDC could help transform these relationships by collaborating with for-profit businesses on health promotion efforts as well as supporting equal partnerships between communities and researchers. These collaborations would deter "helicopter research" — when the researcher flies (or swoops) into a community and collects and publishes data without leaving anything in return (2).

A second recommendation of the panel was to *promote a state-of-the-art e-mechanism to share expertise and knowledge about community health promotion*. Recent research suggests that online information can have a positive impact on consumers' health care. However, for some underserved demographic groups, a "digital divide" (a gap in access to digital information) exists (3), despite numerous technologic initiatives to reduce this gap. CDC could provide leadership by identifying the characteristics and needs of digitally underserved populations and allocating adequate resources to address the identified needs. CDC's role could include engaging nontraditional partners, such as information technology corporations and the U.S. Department of Commerce's National Telecommunications and Information Administration, in efforts to initiate new strategies for helping populations gain access to information. More people could then participate with their providers in making important decisions about their health and well-being.

Increasing evidence shows that to effectively combat current health problems, focusing solely on changing individual behavior is not sufficient; broader determinants of health must also be addressed (4). The expert panel echoed this point and recommended to *shift a measurable part of NCCDPHP's* (National Center for Chronic Disease Prevention and Health Promotion's) *community health promotion programs to focus on improving living conditions across the lifespan* in local communities. However, the panel also recognized that federal health policies do not accommodate this perspective, and therefore recommended that CDC funding be tailored to include a focus on living conditions in communities. Many communities are tackling social, economic, and political issues in an effort to improve their residents' living conditions. However, they are under constant pressure to obtain funding or cease such initiatives altogether. CDC may be able to aid this work in communities by seeking formal agreements with agencies focused on improving people's living conditions (e.g., U.S. Department of Labor, U.S. Department of Housing and Urban Development, U.S. Department of Education).

It will take years for community-based interventions to demonstrate that improving living conditions will change behaviors and improve health among residents. As communities address broader social determinants of health, CDC could work with them to measure intermediate outcomes in order to capture the process by which behavior change is accomplished. If communities are held accountable for demonstrating effective public health interventions, then public health leaders and agencies must also be responsive to the realities these communities face as they address complex health problems today and in the future.
